# Frameless Image-Guided Radiosurgery for Multiple Brain Metastasis Using VMAT: A Review and an Institutional Experience

**DOI:** 10.3389/fonc.2019.00703

**Published:** 2019-08-07

**Authors:** Samir Abdallah Hanna, Anselmo Mancini, Alisson Henrique Dal Col, Rie Nadia Asso, Wellington Furtado Pimenta Neves-Junior

**Affiliations:** Radiation Oncology Department, Sírio-Libanês Hospital, São Paulo, Brazil

**Keywords:** radiosurgery, stereotactic, SRS, VMAT, brain metastases, linac, IGRT, frameless

## Abstract

We undertook a structured review of stereotactic radiosurgery (SRS) using linear particle accelerator (linac) equipment, focusing on volumetric modulated arc therapy (VMAT) technology, and frameless image-guided radiotherapy (IGRT), for the treatment of brain metastases. We analyzed the role of linac SRS and its clinical applications, exploring stereotactic localization. Historically, there was a shift from fixed frames to frameless approaches, moving toward less invasive treatments. Thus, we reviewed the concepts of VMAT for multiple-target applications, comparing its dosimetric and technical features to those of other available techniques. We evaluated relevant technical issues and discussed the planning parameters that have gained worldwide acceptance to date. Thus, we reviewed the current literature on the clinical aspects of SRS, especially its main indications and how the advantages of VMAT may achieve clinical benefits in such scenarios. Finally, we reported our institutional results on IGRT-VMAT for SRS treatments for patients with multiple brain metastases.

## Introduction

Stereotactic radiosurgery (SRS) is a type of external conformal radiation therapy that uses special equipment to tridimensionally position the patient with higher precision than conventional methods, enabling the accurate delivery of single large doses of radiation to small tumors (1). This treatment uses a large number of coplanar or non-coplanar beams or multiple arcs that sequentially irradiate the target to produce a concentrated dose in the lesion while achieving steep dose gradients outside the treatment volume.

The mechanism of SRS differs from that of conventionally fractionated radiotherapy, since the high single doses promote ablation and necrosis of the irradiated target; thus, SRS requires small margins, special planning techniques, and equipment to achieve high conformity and avoid complications (1–3). It is used to treat brain tumors and other brain disorders that cannot be treated by regular surgery. Later, SRS was expanded to also include single-fraction treatments of spinal lesions and then to include fractionated high-dose treatments (4)—also referred to as stereotactic radiotherapy (SRT). SRS concepts and its technical refinements lead to the development of stereotactic body radiotherapy (SBRT), also referred to as stereotactic ablative radiotherapy (SABR), which is the delivery of such complex, accurate and high-dose treatments to extracranial targets (5).

Despite its broad and sometimes confusing definition (6), the term “SRS” is usually reserved for the treatment of intracranial lesions with a single fraction—as first described by the Swedish neurosurgeon Leksell (7) in 1951. In the current American College of Radiology—American Society for Radiation Oncology (ACR-ASTRO) Practice Parameter for the Performance of Stereotactic Radiosurgery, SRS is defined as follows: “For the purpose of this document, SRS is strictly defined as radiation therapy delivered via stereotactic guidance with ~1 mm targeting accuracy to intracranial targets in 1–5 fractions” (4).

A more recent technology, called volumetric modulated arc therapy (VMAT) (8), has features of intensity-modulated radiotherapy (IMRT) as well as arc therapy and therefore produces dose distributions highly adapted to the target volume. This technology appears to be an option in the treatment of multiple brain lesions using a single isocenter (9, 10). The objective of this review is to discuss the specific scenario of treating multiple targets using SRS with VMAT.

## Linac-Based SRS and Clinical Application

Linear accelerator (linac)-based SRS may be delivered using either circular cones or micro-multileaf collimators (MLC) attached to the head of the linac to adjust the aperture through which the target volume is irradiated. The technique with circular cones is particularly useful for treating small and spherical lesions. This technique employs multiple non-coplanar arcs to form a spherical or ellipsoidal dose distribution. For large and irregular targets, it may be necessary to use multiple isocenters per lesion, increasing the dose inhomogeneity and treatment time.

Compared with cones, MLC-based SRS has been shown to produce better dose conformity and reduced treatment time when used to treat larger lesions (1). MLC consists of a computer-controlled array of leaves that can be moved individually to create an aperture, which is dynamically adapted to the target shape. In this modality, the treatment can be delivered as fixed beams or dynamic arcs, named three-dimensional conformal radiation therapy (3D-CRT) and dynamic conformal arc therapy (DCAT), respectively. The MLC also allows the use of intensity-modulated radiosurgery (IMRS), which is the delivery of radiosurgery dose to the patient through several static fields with non-uniform radiation fluency (11). This technique can produce complex dose distributions and is more advantageous for large and irregular tumors.

**Figure 1:**
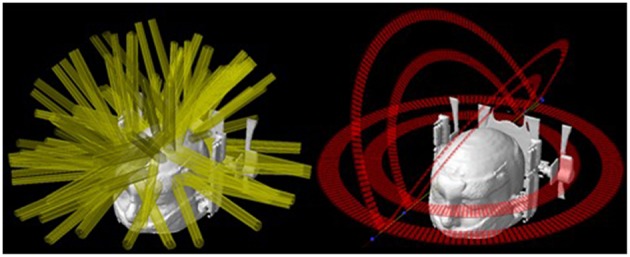
Field arrangements for two treatment techniques. **(Left)** 3D-CRT with 8 isocenters and 62 beams. **(Right)** VMAT with 1 isocenter and 6 arcs.

**Figure 2:**
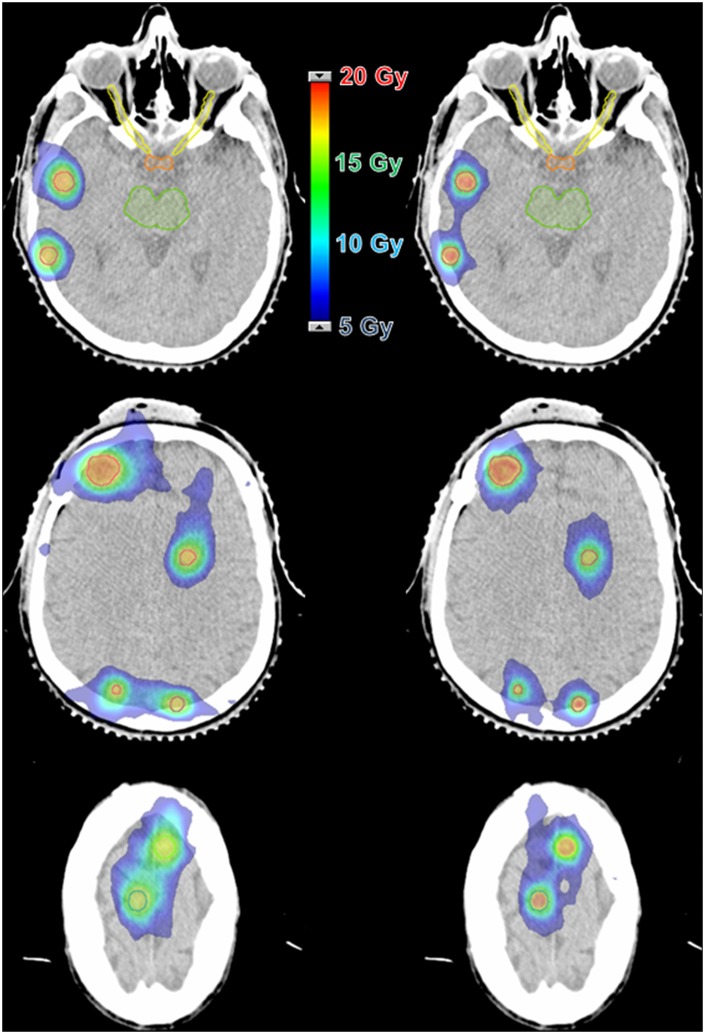
Dose distribution comparison between two techniques for a case with 8 lesions and a prescription dose of 16 Gy. **(Left)** 3D-CRT with 8 isocenters and 62 beams. **(Right)** VMAT with 1 isocenter and 6 arcs.

The major application of SRS is to treat brain metastases, whether in addition to whole-brain radiotherapy, in a post-operative scenario, or as the first treatment. SRS is already a well-established technique for patients with up to three lesions (12, 13), and are promising data for scenarios with more than three and up to ten lesions. The treatment of multiple brain metastases has been a challenging procedure because each one, traditionally, is treated individually. This means that each target needs to be planned to use one (or more) isocenter and several arcs with cones, conformal beams, or dynamic arcs with MLC or Gamma Knife™ (GK) (Elekta, Crawley, UK) shots—depending on the available technology (1, 14). Therefore, the time to accomplish such a procedure is dependent on the total number of targets. Considering that a reasonable time to treat one target is ~20 min, the total time for multiple lesions can take hours to be done. Almost the same difficulties apply to the planning steps for this type of treatment, where each target's plan must be carefully built, calculated and tested. Nevertheless, there is an additional source of complexity: the planner must consider potential contributions of one lesion's plan to the others—and it becomes more difficult with the increase in the number of targets. Figure 1 illustrates the complexity of an SRS plan treating 8 lesions with individual isocenters using static conformal beams compared to a single-isocenter VMAT plan. The resulting dose distributions for each plan are presented in Figure 2.

## Stereotactic Localization—From Fixed Frames to Frameless Approaches

The core difference between SRS and conventional radiotherapy methods is the use of a stereotactic technique, in which the location of a target is related to a three-dimensional Cartesian coordinate system (7). Based on this concept, any intracranial location can be identified in relation to the frame, which was traditionally fixed to the patient's head using sharp pins against the skull. These minimally invasive frames play a role in both positioning and immobilizing patients and are considered effective, with reported accuracy better than 1 mm (2, 15, 16). However, such frames have many drawbacks, including patient anxiety, pain associated with frame fixation, and risk of bleeding and infection at the site of placement (17). If the frame is not properly placed, there is also a risk of movement or slippage (16). Finally, the entire process from simulation to treatment delivery needs to be concluded in a short time (~8 h), since the patient must remain with the frame the entire time. This factor applies stress and pressure to the clinical team, who must complete the tasks as quickly as possible. Thus, depending on the number of lesions, the treatment may become unfeasible.

Advances in image-guided systems allow the development of frameless approaches (15, 16, 18, 19). These make use of non-invasive relocatable immobilizers (examples include precision thermoplastic masks, upper-jaw fixation molds, ear-canal-based positioners, and biteplates) combined with image-guided tumor localization techniques and intrafraction monitoring. The reported spatial accuracy (immobilization and positioning) for such methods are comparable to those achieved with traditional invasive alternatives (17, 20, 21). Although there is no randomized trial comparing those methods, the clinical outcomes are promising, showing an increased level of patient comfort (22–26). Logistically, the use of frameless approaches brings much more flexibility to the planning process, allowing more time to the whole team and even enabling the use of more complex delivery techniques such as VMAT (8).

## VMAT for SRS of Multiple Targets

VMAT is a relatively new technique that allows the delivery of complex dose distributions to a volume in an efficient fashion in one or multiple modulated arcs. The RapidArc (Varian Medical Systems, Palo Alto, CA, USA) is an example of a commercial implementation of the VMAT, based on the work of Otto (8). The RapidArc technique produces highly conformal dose distributions by varying dynamically and simultaneously the dose rate, gantry rotation speed, MLC aperture shape during (up to) 360° arcs.

The feasibility of using VMAT as a delivery option for SRS on multiple metastases using a single isocenter has been demonstrated by Clark et al. (9). This means that the total treatment time becomes independent of the number of targets. The clinical application of this technique can drastically reduce the total treatment time, offering not only efficiency but also a major improvement in patient comfort. Nevertheless, the safe use of the VMAT technique for producing such complex volumetric dose distributions requires extensive dosimetric validation (27).

**Figure 3:**
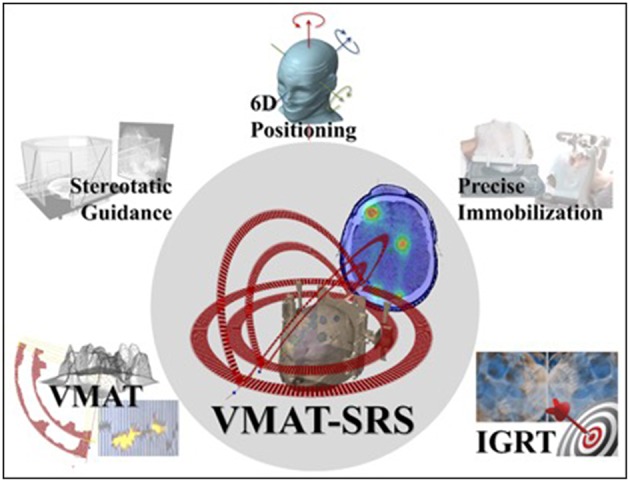
VMAT for multiple-metastasis SRS and the technological tools for its successful clinical deployment.

Additionally, targeting multiple lesions simultaneously has an increased risk of geometrical miss (28, 29). Patient localization errors usually involve both translational and rotational deviations. For single—and usually spherical—lesions placed at the isocenter, small rotational deviations do not play a significant role in treatment delivery accuracy. However, the farther a particular target is from the isocenter, the more displaced it will be from its planned position due to a given rotational deviation. In order to irradiate multiple targets correctly, it is highly desirable to account for both translational and rotational positioning errors. Thus, the successful use of VMAT for multiple brain metastases depends on combining different technologies (Figure 3).

Regarding the quality of the dose distribution in terms of conformity and healthy tissue sparing, although the non-coplanar planning shape is the most commonly used geometry, early comparisons between coplanar VMAT vs. GK for SRS on multiple metastases conducted by Ma et al. (30) found that the volumes of normal brain receiving 4 and 12 Gy were higher for the coplanar VMAT. However, it is clear that any coplanar beam geometry is much more limited than non-coplanar in terms of producing compact dose distributions, especially for lower doses due to the more limited number of beam paths. Later, Thomas et al. (10), using an arguably more refined non-coplanar VMAT planning technique, achieved equivalent dose distributions to those obtained by (GK). Thus, the differences do not seem to be clinically significant, and the dosimetric differences are still matter of debate (30–34).

## Dosimetric and Technical Characteristics

### Is VMAT the Best Choice?

Stereotactic 3D-CRT, DCAT, IMRT, and GK techniques have been used in treating brain lesions for years. However, when treating multiple lesions, these techniques become too time consuming. Single-isocenter VMAT for multiple metastases seems to be equivalent to those techniques in plan quality while requiring less time.

Huang et al. (35) studied 17 patients with 2–5 brain lesions. For patients treated with DCAT/3D-CRT, VMAT plans were retrospectively created, and vice versa. The conformity index (CI) and coverage quality were superior or equivalent for VMAT plans. The mean number of monitor units (MU) decreased by 42%, and the treatment time was reduced by 49%. However, the volume receiving 5 Gy 46% was larger for VMAT. Considering the treatment time, target coverage quality and dose conformity, single-isocenter VMAT seems to be advantageous in multiple brain metastases.

Audet et al. (36) studied 12 patients with cranial tumors with planning target volumes (PTVs) ranging from 0.1 to 29 cm3 and 2 multiple metastases. The plans were performed with RapidArc™ (1–6 non-coplanar arcs), DCAT (~4 arcs), and IMRT (9 static fields). The mean CI for all plans was best for 4 non-coplanar arc VMAT (0.86). The volumes of healthy brain receiving at least 50% of dose prescription were the lowest for the same arc configuration of VMAT and for DCAT. The authors conclude that for lesions similar to those cited in this work and having a diameter larger than 7 mm, VMAT with multiple non-coplanar arcs provides accurate and high-quality radiosurgery with low doses to healthy brain tissue and high dose conformity to the target, as well as the aforementioned time optimization.

Roa et al. (37) studied 16 patients treated with SRS or SBRT through IMRT and VMAT plans with 1 and 2 arcs. Dosimetric conformity, organs at risk (OAR) sparing and homogeneity were similar among the three techniques. The mean beam-on time was reduced by 73%, and MU was reduced by 43%. Since large treatment delivery time increases the probability of intrafractional errors, RapidArc™ seems to be useful in the delivery of SRS.

When comparing the plan quality of VMAT and GK plans, one can infer that both yield somewhat similar results. For example, Liu et al. (34) investigated 6 patients with 3 and 4 brain metastases (volume range 1.70–11.14 cm3) based on plans with 4 to 6 non-coplanar partial arcs. For RapidArc™, the CI value was smaller than for GK (1.19 ± 0.14 vs. 1.50 ± 0.16, *p* < 0.001), and the gradient index (GI) was significantly higher (4.77 ± 1.38 vs. 3.65 ± 0.98, *p* < 0.01). The constraint V_12Gy_ for healthy brain was similar (*p* = 0.58), as were doses such as V_6Gy_, V_4.5Gy_, and V_3Gy_. GK had better results in doses <3 Gy (spread doses). In addition, GK treatment time is 3–5 times longer than VMAT. Furthermore, Thomas et al. (10) conducted 28 treatments of multiple metastases that received a prescription dose of 18 Gy. For the evaluation, 4 non-coplanar arcs was set as the optimal VMAT geometry. Thus, compared with GK, VMAT improved the median CI (1.14 vs. 1.65, *p* < 0.01), and no statistically significant difference was found in median dose fall-off (*p* = 0.269), V_12Gy_ (*p* = 0.500) and low isodose spill (*p* = 0.490).

Therefore, because of relatively low time requirements and similar dosimetric results to the aforementioned techniques, image-guided SRS-VMAT plans seem to be a powerful tool for treating multiple brain metastases with a single isocenter.

### Coplanar vs. Non-coplanar

Once all these techniques were established to be accurate in delivering doses in SRS, linacs performing VMAT plans were revealed to be equivalent. Studies have been conducted to assess the benefits of using coplanar and non-coplanar arcs. Thomas et al. (10) started their article based on the assumption that single-isocenter SRS-VMAT plans are comparable to GK (multi-isocenter technique) considering only 4 non-coplanar arcs, which showed plan quality superior to 1 coplanar arc and 2-non-coplanar-arc plans. Moreover, Clark et al. (9) evaluated the feasibility of single-arc/single-isocenter, triple-arc (non-coplanar)/single-isocenter and triple-arc (coplanar)/triple-isocenter geometries for simulated patients with three near brain metastases. Multiple non-coplanar arcs presented slight improvements in dose conformation to the PTV compared with the other arc geometries.

Lau et al. (26) evaluated 15 patients undergoing SRS-VMAT for multiple targets using a single isocenter. A quantity of 1–4 arcs was used (coplanar except for 4 patients). The median total target volume was 8.3 cm^3^ (range 1.9–93.7 cm^3^), and the median number of tumors was 3 (range 2–13). As a result, the median Radiation Therapy Oncology Group conformity index (RTOG-CI) was 1.15 (range 0.29–2.04), and the median V_12Gy_ was 38 cm^3^ (range 8–432 cm^3^).

Our institution experience (38) is entirely based on multiple and non-coplanar arcs (plans are created at 3–4 couch angles of ~0°, 60°, and 300°). One can see that the median degree of conformity is similar to those reported by the previously mentioned studies: 1.20 (range 0.69–3.14). However, the median V_12Gy_ is better (21.40 cm^3^–ranging from 2.12 to 87.60 cm^3^), considering that our sample also has a similar median total PTV volume (PTV volume summation per patient) of 12.06 cm^3^ (range 0.89–65.05 cm^3^) and that the median number of lesions is 3 (range 2–19).

The bottom line is that non-coplanar single-isocenter arc plans for the treatment of multiple brain lesions seem to be more advantageous (in terms of conformity and sparing of healthy brain tissue) than coplanar arcs.

### Number of Arcs

The hypothesis of treating multiple lesions (single isocenter) with more than one arc may be rational. There is evidence that planning with triple-arc geometry shrinks the volume receiving 12 Gy in healthy brain compared with just one arc (9). Even with the dependence on total PTV volume and lesion number, this constraint (V_12Gy_ < 10 cm^3^) was not infringed, in majority, when compared to GK plans [*p* = 0.51; studies performed by Thomas et al. (10)]. Furthermore, a comparison between 2 coplanar arcs and 3 arcs (which the 3rd partial arc is located vertically) was performed by Wang et al. (39) and it was demonstrated that although the 3-arc plan showed better conformity, it resulted in slightly higher doses of healthy brain, brainstem and chiasm. Perhaps this degradation can be overcome by adding more arcs than just one-half arc, once it was already cited that good results were achieved by other authors. Yuan et al. (40) consider in their work that non-coplanar multiple-arc geometry is superior to coplanar. In this way, 2- and 4-arc geometries were compared, as shown in Table 1.

**Table 1:** Arc geometry set by Yuan et al. (40) for SRS-VMAT (single-isocenter) plans on treatment of multiple brain metastases.

**Arc**	**Plan**	**Gantry start angle (^**°**^)**	**Gantry stop angle (^**°**^)**	**Gantry rotation direction**	**Table angle (^**°**^)**
1	2-arc, 4-arc	181	179	Clockwise	0
2	2-arc, 4-arc	181	10	Clockwise	90
3	4-arc	10	181	Counterclockwise	45
4	4-arc	179	350	Counterclockwise	315

For doses up to 2.8 Gy, 4-arc geometry supports the assumption that more normal brain volume was irradiated with doses up to this level than 2 arcs. However, less healthy brain volume received more than 2.8 Gy with 4-arc than 2-arc geometry.

Audet et al. (36), on the study of non-coplanar arcs for cranial radiosurgery evaluated up to six non-coplanar arcs. The Paddick conformity index (Paddick-CI) (41) for 4 non-coplanar arcs (the best geometry) was 0.86. Also, the volume of healthy brain receiving 50% of the dose prescription was 1.9 times lower than using a single non-coplanar arc geometry. One can summarize that the mentioned arc geometry must be employed to obtain such high-quality SRS-VMAT plans for treating multiple lesions with one isocenter.

Based on our institutional experience (38), SRS-VMAT plans (single isocenter) for multiple targets are performed up to 6 arcs and from 3 to 4 couch angles: 1–2 full arcs at 0°, 4 partial arcs at couch angles around 60° and 300°. For dose prescriptions of 17, 18, and 20 Gy, our (RTOG-CI) and V_12Gy_, as mentioned before, are comprised inside the interval of values reported by the literature, considering the number of tumors, and total target volume. In addition, the mean door-door treatment time was 42 min (ranging from 21 to 62 min), with no correlation with the number of tumors (*R*^2^ = 0.038), but it seems to be correlated with the number of arcs (*R*^2^ = 0.959). It is easy to infer that the more arcs in the plan, the more time is expended during treatment. However, although there is time dependence with the number of arcs, the time expended by SRS-VMAT plans is smaller than the other techniques aforementioned.

### Single or Multiple Isocenters

VMAT planning of multiple targets with a single isocenter has been taking a relevant role in medical physics due its practicability and plan quality (9, 37, 39). One of the comparisons performed by Clark et al. (9) based upon triple arc (non-coplanar)/single isocenter and triple arc (coplanar)/triple isocenter. The V_12Gy_ remained the same for both, but for a single isocenter, small improvements in the CI were observed (this difference might be due to the non-coplanar or single-isocenter geometry as well). All in all, single-isocenter geometry was revealed to be only 50% as time consuming as multiple-isocenter geometry.

Clark et al. (42) studied the plan quality for 1 to 5 lesions based on a single-isocenter VMAT plan. For more than 1 lesion, they recommended 2–4 non-coplanar arcs. The RTOG-CI was (1.12 ± 0.13), and the GI was (3.34 ± 0.42). In addition, Huang et al. (35) enrolled 17 patients with 2–5 brain lesions to evaluate the benefits of this type of geometry for VMAT plans. Among the techniques that use more than one isocenter as DCAT and 3D-CRT, VMAT plans were equivalent to or better than the other two in CI—the authors mentioned that the quality of coverage by VMAT plans was superior and the total treatment time was reduced by 49%.

### VMAT Treatment Planning

Clark et al. (9) used a multiple-arc geometry, limiting the sum of the arc spans up to 1,000°. The Varian High Definition 120 MLC (leaves of 2.5 mm in the centermost 8 cm and 5 mm in the periphery portion of the field) the 6-MV SRS photon beam and a maximum dose rate of 1,000 MU/min were used. The optimization objectives were set to obey the input of *D*_*GTV*_100 = 20 Gy (PTV = GTV [gross tumor volume]) and *D*_*Normal Brain*_ 1% = 10Gy (normal brain excludes the GTV). All in all, the isodose volume that accomplishes 100% of GTV was normalized to 100% dose. The triple arc rotations for single-isocenter geometry were set at couch angles of 0°, 30°, and 330° to produce non-coplanar arcs of (179°-181°), (179°-350°), and (181°-10°), respectively.

Clark et al. (42) published another paper related to SRS-VMAT frameless treatment performed by a 10 MV flattening filter-free (FFF) photon beam at a dose rate of 2,400 MU/min. The paper recommended summing all PTVs into one “PTV_total.” In addition, rings must be created beyond the PTV with the following inner and outer surfaces: 1° ring, 0 mm to 5 mm; 2° ring, 5–10 mm; 3° ring, 10–30 mm. These regions receive 98, 50, and 40% of the prescribed intensity, respectively (each individual target was set to receive 102% of the prescribed intensity in 100% of each target volume). Yuan et al. (40) also conducted some studies based on these planning methodologies as well. However, it was not evident what grid size (GS) was set for the dose calculation.

Karen et al. (43) studied the effect of the GS to evaluate the accuracy of VMAT spine Stereotactic Body Radiation Therapy (SBRT). Although this study was performed over SBRT treatments, the outcomes related to dose-fall-off can be linked to SRS treatments, once both have the conjecture of producing a high dose gradient beyond the targets. GS of 1, 1.5, and 2.5 mm was investigated and evaluated based on distance to fall-off (DTF) between 90 and 50% isodose line, D10% and D0.03 cm3 on spinal cord adjacent to target. Based on 1 mm GS, the DTF increased for 1.5 and 2.5 mm (e.g., 2.52 ± 0.54 mm; 2.83 ± 0.58 mm; 3.30 ± 0.64 mm, *p* < 0.001, respectively); The D10% and D0.03 cm3: 6.24 and 7.81% (for 1.5 mm) and 9.80 and 13% (for 2.5 mm). Therefore, plans calculated with a GS of 1 mm have to be employed in situations where reaching a high dose gradient is aimed.

Based on the assumption of GS, one can discuss the study carried by Hossain et al. (44) who conducted a work with 1 patient possessing 12 lesions to compare the results between SRS-VMAT and GK plans. They concluded that for all VMAT results, the low isodose level volumes of 8- and 4-Gy were averaged (275 ± 132) % higher when compared to GK values. For 12-Gy and 16-Gy, the isodose volumes were approximately (179 ± 91) % and (129 ± 40) % higher than GK, respectively. Once the dose prescription for all targets was 20 Gy, 80, 60, 40, and 20% of the prescribed dose was evaluated. In that way, some uncertainties may add into this and the work conducted by Yuan et al. (40), once they used 2.5 mm of GS in the calculation process. However, Yuan et al. (40) did not compare VMAT with any other technique; only comparison among VMAT arc geometries was performed. Thus, the possible systematic errors associated with the calculus were present for all of them.

Our department's planning routine (38) consists, first of all, of creating a PTV margin of 2 mm from GTV to consider geometrical uncertainties due to the entire treatment process. A volume called “Healthy Brain” was created by subtracting GTV and, around each PTV, two spherical shells were created (the first starts at the PTV borders with 0.5 cm thickness and the second starts at 0.5 cm from PTV border with 2 cm thickness) to achieve steep dose fall-off at the vicinity of PTV. VMAT plans were created to run in a linear accelerator equipped with a high-definition MLC (HD-MLC) with 120 leaves—the innermost 8 cm with 2.5 mm width leaves and the other outermost leaves with 5 mm width, completing 22 cm longitudinal field size (Varian, Palo Alto, USA). The optimization was performed aiming at least 99% coverage of all PTVs with the prescribed dose. The maximum dose inside each PTV was controlled to remain encircled within the GTV. The final calculation was performed with Eclipse Anisotropic Analytical Algorithm (AAA) versions 10–15 with 1 mm of calculation GS.

### Impact of Target Distance From the Isocenter

A common question might arise about how the target distance from the isocenter can negatively affect the plan quality. As mentioned by Clark et al. (42), when utilizing frameless SRS-VMAT plans with a single isocenter designed for the treatment of multiple metastases, care must be taken to guarantee accurate patient positioning. Small rotations can result in a major impact on dose coverage, especially for small lesions.

To address rotation errors impacts over dose delivery accuracy, a 6-degree of freedom (6-DOF) couch and image registration is recommended. Kim et al. (28) evaluated the positional variations of five off-axial metallic ball bearing markers for a single-isocenter SRS-DCAT (once, for this purpose, DCAT carries simple geometric interpretation and can generate the same geometric accuracy results as SRS-VMAT plans). The phantom was immobilized by a frameless thermoplastic mask, and an MLC margin of 3 mm was introduced outward PTV. The ExacTrack™ 6D (BrainLab, Feldkirchen, Germany) patient positioning system was used, and a total positional error for the MLC aperture of 0.61 ± 0.2 mm was found along the rotational path of the arcs employed in this study. In addition, Adamson et al. (45) evaluated the challenges of implementing a single-isocenter SRS-VMAT plan for treating multifocal intracranial disease. They used a thermoplastic mask for immobilization and a VMAT technique with HD-MLC (2.5 mm with innermost leaves). Using 6-DOF positional correction, they concluded that a 1 mm margin was necessary to compensate for spatial uncertainty within the mask. In general, it is a good choice to perform frameless SRS-VMAT plans with 6-DOF couch corrections, considering the respective margins.

Tryggestad et al. (29) evaluated frameless positioning data of patients with brain tumors based on cone-beam computed tomography (CBCT) pre- and post-treatment scans. A set of four immobilization masks was studied to obtain the systematic inter- and intrafraction, as well as the random intrafraction for translation and rotation shifts. By focusing on setup and positioning rotational errors and the selection of a suitable mask, one can observe a systematic interfraction error with a mean (SD) of 0.00° (0.90°), −0.34° (0.80°), and 0.39° (0.90°), for medial-lateral (ML); cranial-caudal (CC); and anterior-posterior (AP) displacements, respectively. Random interfraction errors were 0.6, 0.8, and 0.7, respectively. The random intrafraction positioning error was approximately −0.06° (0.40°), −0.17° (0.5°), and −0.06° (0.60°), respectively. Therefore, once it was possible to handle systematic errors by the On-Board Imager (OBI) (Varian, Palo Alto, USA) such as CBCT or another equivalent, a PTV margin of 0.7 mm could be achieved based on the best thermoplastic mask they studied.

In this context, Clark et al. (46) conducted a work that evaluated the dosimetric effects when PTVs are shifted in 1°, 2°, and 3° each for roll, pitch and yaw, relative to isocenter and maintaining dose distribution constant. The targets had a mean volume of 2.1 cm3 (range 0.1–18 cm3) and a mean distance from the isocenter of 4.2 cm (range 1.0–7.1 cm). It was evident that rotations ≥2° reduced coverage below 95% of PTV volume receiving 95% of prescription. In conclusion, minimizing rotation error below 1° is vital for aimed coverage (especially for small lesions).

In addition, Roper et al. (47) determined the dosimetric effects of rotational errors on coverage as well. Considering ideal values of D95 ≥ 100% and V95 = 100%, they found that at 0.5° rotational error, D95 values and V95 coverage rates were larger than or equal to 95% in all 50 cases. For 1.0°, 7% of targets showed D95 and V95 below 95%. Finally, for 2° rotational error, 47% of the targets lied below 95% for D95 and V95 (consider the mean ± SD in PTV and distance from isocenter of 0.96 ± 1.25 cm3 and 3.53 ± 1.61 cm).

Additionally, treating multiple lesions that are too far away (roughly > 10 cm apart) using a common isocenter brings other planning difficulties related to MLC mechanical limitations that need to be considered, such as MLC maximum field size, leaf span and, in the case of Varian Millennium HD-MLC, the use of outermost thicker 5 mm leaves. There are situations that may require planning maneuvers, such as increasing the number of arcs or even splitting the plan on more than one isocenter.

In summary, margins of PTV must be taken, and an appropriate image-guided radiotherapy (IGRT) program must be employed in all institutions that aim to treat multiple lesions distant from the isocenter in a single-isocenter VMAT technique.

## Clinical Aspects

### SRS Indication for Multiple Brain Metastases

Several Phase III studies (48–50) and meta-analyses support SRS and/or surgical resection as initial treatment for patients with few brain metastases compared to whole-brain radiotherapy (WBRT). The main benefits include non-inferiority in median survival, local control, and a decrease in the likelihood of having cognitive decline.

These findings can be exemplified by some trials, such as the individual patient data meta-analysis well-conducted by Sahgal et al. (51), where they analyzed 3 pivotal Phase 3 trials [Aoyama [JROSG] (13), Kocher et al. (49) [EORTC 22952- 26001] and Chang et al. (52)], among others, involving patients with one to four brain metastases. They could correlate the age (50 years as cut-off) and the number of lesions (one as cut-off) as effect-modifiers for treatment. They also showed that the risk of mortality [HR: 0.72 (0.57–0.90)] and distant brain failure [HR: 0.63 (0.46–0.88)] were significantly higher in patients with 2 or more lesions. For patients ≤50 years and carrying one lesion, the overall survival was significantly better in the SRS group.

Notwithstanding, there are Phase III trials hypothesizing that SRS alone might be appropriate for patients with more than 4 lesions. In this context, upfront SRS or even SRS as salvage therapy after initial treatment (surgery or SRS) may be adequate for those patients (50, 53). The outcomes will be the maintenance of overall survival while avoiding the neurocognitive impairment caused by WBRT. Nonetheless, one may argue that WBRT also eradicates microscopic disease, which is not possible with SRS alone, and may be more cost effective than SRS (54)—the patients may not need further surgery or SRS, and they will not need to undergo control resonances quite as often. Furthermore, once multiple lesion treatment with SRS is performed, the difficulty of re-irradiating recurrent lesions increases because of the cumulative dose.

In a prospective observational non-inferiority trial conducted by Yamamoto et al. (55), Gamma Knife was applied to patients with up to ten brain tumors. They have observed that SRS with five to ten lesions compared with patients carrying one to four brain metastases were equivalent. The median overall survival were 10.8 months (*p* = 0.78; *p*_*non*−*inferiority*_ < 0.0001) and same percentage of treatment-related adverse event (9%, *p* = 0.89). Secondary outcomes like neurological death, local recurrence, repeat SRS for new tumors also maintained equivalent (56).

There might be some reasons for why patients with multiple tumors are unsuitable for SRS treatments beyond clinical features, such as neurocognitive decline. One of them is indeed the treatment time (10). Considering the time spent for each isocenter being 20–30 min, many patients are not supposed to be able to remain still for more than 30 min on the linac couch. Consequently, there must be necessary bringing the patient to radiotherapy facility more than one time to treat several isocenters. Thus, the possibility of treating more rapidly these patients might also improve the radiotherapy facility's workflow (57). The treatment time itself has never been studied as a surrogate for patient adherence, or treatment tolerance, even if in practical terms this situation is common.

There are some studies exploring the role of VMAT on the possibility of incorporating WBRT with protection of sensitive structures, such as hippocampi (58) and cochleae, and concomitantly treat grossly evident lesions with a SRS boost (59).

Since the development of VMAT technology, it has increasingly become one of treatments of choice in the event of brain metastasis, mainly due to its inherent advantages, such as the ability to treat multiple lesions concomitantly in a reduced timespan while maintaining the dosimetric characteristics of SRS. Today, it is possible to classify VRS (volumetric SRS) as such a treatment, according to the American Association of Neurological Surgeons and the Congress of Neurological Surgeons. These groups defined SRS as “high-precision treatment sessions of 5 fractions” (60).

### Clinical and Practical Results

Thus, far the majority of studies on VMAT have primarily discussed dosimetric issues. On the other hand, clinical trials involving the treatment of multiple metastases rarely mention the techniques that are used. Thus, it becomes worth reviewing the literature in hopes to connect these two sides.

The maximum number of metastases that can be treated with SRS is not well-established. Yamamoto et al. (53, 55) analyzed 80 patients with 10 or more metastases, totaling 1,710 lesions (median lesion number: 17 and median cumulative volume: 8.02 cm3). Despite the use of single-fraction radiotherapy, the doses were ~2.60–6.69 Gy in areas far from the targets, and only a small volume of normal brain tissue received high doses. In SRS for multiple lesions, the major drawback is based upon the increase in radiation doses to healthy brain because of the overlap in planning for each target. Therefore, in VMAT, it is possible to optimize all dose distributions in a single plan.

Using the rational benefit of WBRT plus SRS, Lee et al. (61) investigated the clinical application of VMAT for four or more brain metastases in association with WBRT, sequentially (15–30 Gy in 4–10 fractions) or simultaneously (48–50 Gy in 10–20 fractions). This retrospective study demonstrated 12-month overall survival of 41.7%, a median survival time of 9 months, and 12-month local progression-free survival of 62.5%. Although the analysis of late toxicity and marked worsening in cognition was not reported in detail by the authors, the fact that there was no serious neurocognitive deterioration makes it possible to infer that this treatment does not deliver severely damaging doses to healthy brain tissue.

In a similar Canadian study, Nichol et al. (62) analyzed 60 patients with one to ten brain metastases who underwent fractionated treatments (50 Gy in 5 fractions at 95% isodoses, delivered to gross lesions, concomitant to WBRT−20 Gy in 5 fractions). The investigators also used IGRT approaches. At a median follow-up time of 30.5 months, the median survival was 10.1 months, the rates of total and partial brain response were 56%, and the prevalence of local control was 88%. The rate of radionecrosis grades 3–5 was 25% for deeply located tumors and 1.9% for non-deep metastases.

From the point of view of clinical outcomes, there is no level I evidence supporting the use of SRS with VMAT compared to other techniques. The most robust related information is the multivariate analysis from the study of Andrews et al. (12), which compared clinical outcomes between Gamma Knife and linacs in the setting of SRS plus or minus WBRT for brain metastases, showing that there were no differences.

In a study by Lau et al. (26) at the University of San Diego, single-isocenter frameless VMAT was performed in 15 patients, with a median dose of 20 Gy in a median of 3 brain metastases. The median follow-up time was 7.1 months. At 1 year, local and regional control were achieved in 81.5 and 60% of cases, respectively, and the overall survival was 39%; there was no treatment-related toxicity of grade 3 of higher. The investigators also reported a mean dose to normal brain of 4.2 Gy, median V_12Gy_ of 38.0 cm^3^, and median V_4.5Gy_ of normal brain of 350.5 cm^3^. No discernible relationship between the dose to normal brain tissue and the degree of toxicity was observed.

Another study addressing the role of VMAT for multiple lesions was conducted by Fiorentino et al. (63), where they analyzed early clinical results in 45 patients treated with linac-based SRS/fractionated stereotactic radiotherapy (FSRT) FFF delivery using VMAT. The prescribed dose ranged from 15 Gy single-shot treatment to hypofractionated treatments (5 fractions). The authors included patients with up to 5 brain metastases and carrying good performance status. The mean “beam-on” time ranged from 90 to 290 s for each lesion. Their median follow-up was 12 months, where the local control achieved 93.2%, and the median overall survival reached 77%. In addition, they could not observe severe adverse events.

At our institution (64), we started using VMAT for multiple brain tumor treatments in 2012, after some specific training of our staff. Through this time, we evaluated 32 patients with a mean age of 61 years and 4 lesions per treatment (1–19), accounting for 141 lesions undergoing SRS with VMAT, of whom 28 lesions were treated with single-shot radiosurgery. We started expanding 2 mm the lesions toward PTV margins for any uncertainties, even though our quality controls would guarantee intrafraction errors of <1 mm. Only 5 cases presented with single lesions. The mean tumor volume per patient was 15.9 cm^3^ (ranging from 0.89 to 74.70 cm^3^). The medium follow-up time was 5.6 months. There were 12 brain recurrences (3 local and 9 diffuse). Five patients progressed with leptomeningeal disease, and 13 had distant disease progression. Regarding toxicity, 2 patients presented radionecrosis, and only 1 experienced neurocognitive decline. We can conclude by analyzing these findings that our results are compatible with the scarce literature.

## Conclusions and Remarks

It is worth analyzing from the clinical point of view all the dosimetric advantages observed in all comparisons of VMAT with other cranial radiosurgery techniques, especially in the context of multiple lesions. As explained above, VMATs are similar to the “non-VMAT” approach in their conformality and heterogeneity; additionally, the possibility of concomitantly treating multiple lesions is attractive; and finally, the IGRT system allows frameless treatments.

Unfortunately, the medical literature is scant regarding clinical outcomes of VMAT use in the context of initial SRS treatments. The available references show few results, with few patients and only preliminary follow-up.

The state of this field can be discouraging, but we believe there are two important issues to be highlighted. First, perhaps the VMAT technique is now developed enough in terms of dosimetric safe which may render unnecessary any randomized studies that exclusively compare one technology against the other. The clinical advantages, such as the possibility of concomitant treatment of multiple lesions with safety and effectiveness, need not be directly tested. Second, the absence of robust clinical trials exclusively using VMAT as a therapeutic modality will continue to encourage scientists to seek the most useful evidence to support physicians.

Finally, given the existing dosimetric research on the safety and benefits of VMAT, there is an ample basis to indicate this technology as a therapeutic modality of SRS. Our institution has initiated this approach in patients with multiple metastases, and we are currently implementing this option without encountering any adverse events. Hastening treatment will undoubtedly impact patients' quality of life.

## Ethics Statement

The retrospective dosimetric data collection of institutional plans was carried out with approval of Human Research Ethics Committee of the Sírio-Libanês Hospital (study 962). The study is also registered in the Brazilian Platform of Human Research (CAAE: 12157319.9.0000.5461).

## Author Contributions

All authors contributed equally to the design and revision of the article. AD, AM, and WN-J contributed to the physics parts, whereas RA and SH prioritized the clinical parts. SH conducted the final review.

### Conflict of Interest Statement

The authors declare that the research was conducted in the absence of any commercial or financial relationships that could be construed as a potential conflict of interest.

## Abbreviations

AAA: anisotropic analytical algorithm
AAPM: American Association of Physicists in Medicine
ACR-ASTRO: American College of Radiology, American Society for Radiation Oncology
CBCT: cone-beam computed tomography
CI: conformity index
DCAT: dynamic conformal arc therapy
DTF: distance to fall-off
EORTC: European Organization for Research and Treatment of Cancer
FSRT: fractionated stereotactic radiotherapy
GK: Gamma Knife
GS: grid size
GTV: gross tumor volume
Gy: Gray
HD-MLC: high-definition multileaf collimator
IGRT: image-guided radiation therapy
IMRS: intensity-modulated radiosurgery
IMRT: intensity-modulated radiotherapy
JROSG: Japanese Radiation Oncology Study Group
Linac: linear accelerator
Min: minute
MLC: multileaf collimators
MU: monitor unit
MV: megavoltage
OBI: on-board imager
PTV: planning target volume
RTOG: Radiation Oncology Group
SABR: stereotactic ablative body radiotherapy
SBRT: stereotactic body radiation therapy
SRS: stereotactic radiosurgery
SRT: stereotactic radiotherapy
VMAT: volumetric modulated arc therapy
VRS: volumetric radiosurgery
WBRT: whole-brain radiotherapy
3D-CRT: three-dimensional conformal radiation therapy
6-DOF: 6-degree of freedom.
